# Evaluating the Potential of *Piper carpunya* Extracts: Antimicrobial and Antioxidant Effects in a Risotto Food Model

**DOI:** 10.3390/foods15152762

**Published:** 2026-08-06

**Authors:** Jorge F. Reyes, Iván Burneo, Ana M. Diez, Beatriz Melero, Jordi Rovira, Isabel Jaime

**Affiliations:** 1 Departamento de Química, Universidad Técnica Particular de Loja, San Cayetano Alto s/n, Loja 1101608, Ecuador; 2 Carrera de Ingeniería Ambiental, Facultad Agropecuaria y de Recursos Naturales Renovables (FARNR), Universidad Nacional de Loja, Loja 110150, Ecuador; ivan.burneo@unl.edu.ec; 3 Department of Biotechnology and Food Science, University of Burgos, 09001 Burgos, Spain; amdiez@ubu.es (A.M.D.); bmelero@ubu.es (B.M.); jrovira@ubu.es (J.R.); ijaime@ubu.es (I.J.)

**Keywords:** *Piper carpunya* extract, antimicrobial activity, pathogenic bacteria, spoilage bacteria, risotto

## Abstract

This study evaluated the antimicrobial properties of *Piper carpunya* extracts, which are traditionally used by Ecuadorian indigenous communities, and validated the in vitro findings in a real food matrix. Three different extracts (ethanolic, hydroethanolic, and aqueous-lyophilized) were evaluated by the agar well diffusion method against a panel of 20 microbial strains comprising both foodborne pathogens and spoilage microorganisms. The extracts with the highest yield and antimicrobial activity were then tested in a precooked risotto formulated under commercial conditions, in challenge tests with *Clostridium perfringens*. All extracts inhibited *C. perfringens* at a minimum inhibitory concentration below 80 mg/mL, the ethanol extract being the most effective. In the risotto model, the ethanol–water extract (8%, *w*/*w*) reduced *C. perfringens* counts by approximately 2 log CFU/g relative to the inoculated control and also lowered aerobic mesophilic and psychrophilic counts, whereas the freeze-dried extract was less effective and bacterial counts declined only until day 4 of storage. Both extracts slightly reduced pH and water activity and, rather than protecting the lipid fraction, exhibited a prooxidant effect; nevertheless, malondialdehyde remained below 0.35 mg/kg throughout the 30-day storage period, well below the thresholds associated with perceptible rancidity. *P. carpunya* extracts show potential as natural ingredients for improving the microbiological safety of ready-to-eat foods, and their chemical and sensory characterization is required before practical application.

## 1. Introduction

The food industry is currently striving to meet consumer preferences for minimally processed products, prioritizing safety without the use of synthetic additives [1,2,3]. In this context, food processors have recognized that “clean label” products satisfy these, and they regard such products as a key market opportunity [1,4,5]. It should nevertheless be acknowledged that this definition is subjective and is predominantly associated with three fundamental elements: a short list of ingredients, trust in those ingredients, and the perception of healthiness [6,7].

Against this background, the use of plant-derived extracts [8]—obtained from fruits, spices, and herbs rich in phenolic compounds [9]—and of terpene-rich essential oils [7] is of considerable interest. The growing demand for these products can be attributed to their capacity to replace artificial ingredients, which consumers frequently regard as detrimental to their health [6]. However, challenges remain, particularly with regard to their cost, stability, and availability [10]. Such extracts are used in a variety of applications, including coloring, flavoring, and preservation [11]. Research has shown that the antioxidant and antimicrobial capacities of certain natural extracts can extend the shelf life of foodstuffs, a property associated with high concentrations of polyphenols, alkaloids, terpenoids, and tannins, among other compounds [7,12,13].

In countries such as Ecuador, which is characterized by remarkable biodiversity [13,14] and large markets for medicinal plants [15,16], the use of plant-derived materials [17] as alternatives to conventional food preservatives is of considerable economic importance. Many species of the Ecuadorian flora remain largely uninvestigated despite their historical documentation in traditional medicine and culinary practices [18,19]. One such species is *Piper carpunya* Ruiz & Pav. (Piperaceae), which is used either fresh or in the preparation of a traditional beverage known as *Guaviduca* [18], that is reputed to possess therapeutic properties against ailments such as colds, gastrointestinal disorders, and dermatological conditions [19]. Several studies have examined the gastroprotective, anti-inflammatory [20], and anti-*Helicobacter pylori* [21] properties of the natural compounds present in *P. carpunya* extracts.

*P. carpunya* has been shown to contain a variety of bioactive molecules with anti-microbial activity [21]. Notable among these are flavonoids such as vitexin, isovitexin, and rhamnopyranosylvitexin, which are recognized for their potent antioxidant and antimicrobial properties. Terpenoids such as sitosterol and stigmasterol are also antimicrobial, in addition to their gastroprotective benefits. The neolignan (6S, 9S)-roseoside exhibits potent antibacterial activity, particularly against *Staphylococcus aureus* and *Bacillus subtilis* [21]. In addition, the dihydrochalcone asebogenin has been identified as a notable agent with antifungal [22], antibacterial [21], antimalarial [23], and antithrombotic [24] properties.

*P. carpunya,* together with various other species of the genus *Piper*, has been shown to exhibit antimicrobial activity, thereby inhibiting the proliferation of bacterial strains such as *Klebsiella pneumoniae, Staphylococcus aureus and Escherichia coli* [25], *Pseudomonas aeruginosa*, *Bacillus subtilis*, and *Helicobacter pylori,* as well as fungal species such as *Candida albicans* [18,25,26,27,28]. Despite these in vitro findings, the transition of these extracts into real biological models remains a developing field. One notable practical application of *P. carpunya* is the use of its ethanolic extract to suppress the development of the fungus *Sphaerotheca pannosa* var. *rosae* [29]. Furthermore, its essential oils have shown high efficacy against critical cacao pathogens, including *Moniliophthora roreri*, *Lasiodiplodia theobromae*, and *Fusarium ipomoeae*, in several cases achieving complete suppression of mycelial growth and spore germination [30]. Since these pathogens are responsible for the most economically damaging diseases in the commercial rose and cacao industries, products derived from *P. carpunya* emerge as sustainable and effective biocontrol alternatives [29,30]. The antimicrobial and antifungal activities of *P. carpunya* are closely linked to its chemical composition. Analyses show that its essential oils are characterized by a high concentration of phenylpropanoids [30], as well as monoterpenes (such as piperitone, terpinene, and limonene), 1,8-cineole, phytosterols, and flavonoids [21,26,31,32,33]. These constituents have been identified in phytochemical studies of both the leaves and essential oils.

Beyond these largely in vitro observations, several species of the genus Piper have already been assayed in real food systems. The essential oil of *P. nigrum*, for instance, has been applied to sous-vide red deer meat, where it limited the growth of Listeria monocytogenes and of the native microbiota during refrigerated storage [34], and the preservative applications reported for the genus have been compiled in a comprehensive review [35]. Comparable strategies have been described for other plant materials rich in phenolic compounds and terpenoids, including extracts of *Simira ecuadorensis* applied to chicken and fish products [13] and *P. nigrum* fractions assayed against foodborne bacteria [36]. Taken together, these studies indicate that the genus is a credible source of “clean-label” preservatives, but they also show that the available evidence remains fragmented: it covers a small number of species, is dominated by essential oils rather than by solvent extracts, and rarely combines a broad antimicrobial screening with a challenge test performed in a formulated product stored under commercial conditions. For *P. carpunya* in particular, the published work is restricted to pharmacological and phytochemical characterization [21,32] and to agricultural biocontrol [29,30]; to the best of our knowledge, its behavior as a preservative inside a food matrix has not been reported.

The scientific literature addressing the antimicrobial properties of *P. carpunya* is limited to a few bacterial and fungal species. A notable aspect of the present study is that biological activity was verified against a wider range of spoilage and pathogenic bacteria found in foods, and that the effectiveness of the extracts was evaluated once they had been incorporated into food formulations. Consequently, this study sought (i) to evaluate the antimicrobial efficacy of *P. carpunya* extracts against bacterial growth in vitro, and (ii) to assess their feasibility as natural preservatives in a precooked risotto used as a real food matrix, monitoring the microbiological, physicochemical, and oxidative changes that occurred throughout refrigerated storage. The chemical characterization of the extracts was outside the scope of this work and is addressed in a companion study by our group [37].

## 2. Materials and Methods

### 2.1. Raw Materials

#### 2.1.1. Collection and Preparation of Piper carpunya Leaves

Leaves of *Piper carpunya* were collected in Gualel (Loja Province, Ecuador) in November 2021. The collected material was first cleaned and then dehydrated at 40 °C for approximately 12 h in a tray dryer (DY-110H, Daeyeong E&B Co., Ltd., Ansan, Republic of Korea). The dried leaves were subsequently milled, sieved, and thoroughly mixed to obtain a homogeneous powder with a particle size below 350 µm. The resulting material was kept under refrigeration until the extraction procedures were performed.

#### 2.1.2. Preparation of Plant Extracts

The extracts were obtained by static maceration. The solvent-leaves mixture was placed in an amber container and refrigerated for three days, with stirring once or twice daily, and was then vacuum-filtered. Water, ethanol, and a mixture of 50% ethanol and 50% water (by volume) were used as solvents, at a ratio of five liters of solvent per kg of leaves. The ethanolic and hydroethanolic extracts were prepared by evaporating the ethanol in a rotary evaporator (G1, Heidolph, Schwabach, Germany) at 40 °C and 150 rpm. The water was then removed by freeze-drying in a lyophilizer (Labconco, Kansas City, MO, USA) under the following conditions: pressure = 0.180 mbar; temperature = −50 °C for 72 h. These extracts are hereafter referred to as “ETOH” and “ETOH–H2O”, respectively. To obtain the aqueous extract, the filtrate was first stored at −40 °C and then freeze-dried under the conditions described above. The resulting lyophilized powder was designated as “L”. Freeze-drying was selected because it preserves thermolabile bioactive constituents and minimizes their degradation, thereby retaining the functional properties of the extract, including its antioxidant and antimicrobial activities [38,39,40]. The extraction yields of the ETOH, ETOH–H_2_O, and L extracts were 1.71%, 4.04%, and 10.85%, respectively.

### 2.2. Study Design

In the preliminary phase, and owing to the scarcity of available data on the antimicrobial potential of *P. carpunya*, an initial in vitro study was conducted with the primary objective of establishing a comprehensive database. This involved evaluating the antimicrobial activity of the extracts against a collection of 20 foodborne pathogenic and spoilage microorganisms.

Following the initial screening, the extracts were further evaluated for their inhibitory activity against *Clostridium perfringens*. This was accomplished by inoculating the selected microorganism into a model of a food system. For the challenge tests, risotto was selected as the model food because it is a representative medium in which these microorganisms are typically encountered.

### 2.3. Antimicrobial Activity “In Vitro”

#### 2.3.1. Test Microorganisms

A total of 20 bacterial strains were selected for the in vitro antimicrobial assays. These cultures were supplied by the Spanish Type Culture Collection (CECT), the International Life Sciences Institute (ILSI), and the University Hospital of Burgos–University of Burgos (HUBU–UBU). A complete list of the microorganisms included in the study is given in Table 1.

#### 2.3.2. Agar Diffusion Assay

The antimicrobial potential of *P. carpunya* extracts was evaluated using a modified agar well diffusion assay [41]. Bacterial cultures were incubated until they reached approximately 8 log CFU/mL, after which the suspensions were spread uniformly over the surface of solid culture media. Once the agar had solidified, wells 7 mm in diameter were prepared aseptically with a sterile pipette tip. Before testing, each extract was adjusted to a concentration of 80 mg/mL with a 1:1 (*v*/*v*) mixture of deionized water and dimethyl sulfoxide (DMSO). Subsequently, 40 µL of the corresponding solution was dispensed into each well. After incubation for 24–48 h under the culture conditions appropriate for each microorganism (Table 1), antimicrobial activity was assessed by measuring the diameter of the inhibition zones with a caliper. A 1:1 (*v*/*v*) deionized water–DMSO solution served as the negative control, whereas 0.94% sodium hypochlorite was included as the positive control.

Bacterial strains exhibiting susceptibility in the agar diffusion assay were selected for determination of the minimum inhibitory concentration (MIC). The extracts were serially diluted in a 1:1 (*v*/*v*) deionized water–DMSO solution to final concentrations of 80, 40, 20, 10, and 5 mg/mL. The antimicrobial assay was then repeated as described above, and the MIC was defined as the lowest extract concentration that completely prevented visible bacterial growth. All experiments were performed in triplicate.

### 2.4. Challenge Test

The preservative impact of *P. carpunya* extract was evaluated in a precooked risotto, which was chosen as a model food because rice-based products are highly susceptible to microbial growth [42,43,44].

#### 2.4.1. Preparation of *C. perfringens* Inoculum

Two reference strains of Clostridium perfringens (CECT 376 and CECT 563), obtained from the Spanish Type Culture Collection (CECT), were selected for this stage of the study. Each strain was streaked onto nutrient agar supplemented with 5% sheep blood (Oxoid Ltd., Basingstoke, Hampshire, UK) and incubated at 37 °C for 24 h under anaerobic conditions (<0.15% O_2_ and 1–15% CO_2_) generated using the AeroGen^®^ system (Thermo Fisher Scientific, Oxford, UK).

An isolated colony from each *C. perfringens* strain was aseptically transferred to a tube containing 5 mL of Brain Heart Infusion (BHI) broth (Oxoid Ltd., Hampshire, UK). The cultures were incubated overnight under anaerobic conditions. After incubation, the bacterial suspensions were adjusted to approximately 8 log CFU/mL in sterile BHI broth on the basis of optical density measurements. Equal volumes of the two suspensions were then combined (1:1) and diluted with sterile water to obtain a final concentration of 6 log CFU/mL. The resulting inoculum was incorporated into the food samples at a maximum level of 1% (*w*/*w*) to minimize any effect on product characteristics. Control samples received the same proportion of sterile water instead of the bacterial inoculum.

#### 2.4.2. Risotto Product

A precooked risotto model containing 3.5% fat was formulated in order to evaluate the antimicrobial and antioxidant efficacy of *P. carpunya* extracts under food matrix conditions. The formulation was based on the technical specifications of commercially available risotto products and was optimized in preliminary trials. The final composition (per 100 g of product) consisted of 25.0 g round rice (Hacendado, Valencia, Spain), 16.7 g light cooking cream (Hacendado, Valencia, Spain), 27.8 g whole milk (Hacendado, Valencia, Spain), and 30.5 g water. After vacuum packaging in plastic pouches, the samples were cooked in a water bath (Selecta, Barcelona, Spain) at 80–85 °C for 30 min. The selected time–temperature combination ensured adequate cooking of the rice and produced a texture comparable to that of the simulated product. Each treatment was transferred into sterile plastic bags that had been previously exposed to UV irradiation for 15 min, after which 5 g of the food model was added to each bag. For treatments containing *P. carpunya* extract, the extract was incorporated into the cooked risotto at a concentration of 8% (*w*/*w*) before packaging. This level was selected on the basis of the in vitro results: 8% (*w*/*w*) corresponds to approximately 80 mg of extract per gram of product, that is, four times the MIC determined for the ETOH–H_2_O extract against *C. perfringens* (20 mg/mL) and equal to the MIC of the freeze-dried extract (80 mg/mL). Concentrations at or above the in vitro MIC are generally required in food matrices, because interactions of the active compounds with lipids, proteins, and carbohydrates reduce their availability and attenuate their antimicrobial effect [45,46,47].

Six experimental treatments were established for the *C. perfringens* challenge study: C, control (without extract); E, risotto containing the ETOH–H_2_O extract; L, risotto containing the freeze-dried extract; P, inoculated control; EP, inoculum and ETOH–H_2_O extract; and LP, inoculum and freeze-dried extract. Considering that the commercial shelf life of this food product is approximately 30 days under refrigeration, analyses were performed after 0, 1, 4, 7, 15, and 30 days of storage at 5 °C. On day 0, treatments C and P were additionally evaluated to confirm the initial hygienic quality of the samples. At each sampling time, three independent (10 g each) samples from every treatment were collected for microbiological analyses. In addition, three further samples from treatments C, E, and L were used for physicochemical determinations (lipid oxidation, water activity, and pH).

#### 2.4.3. Microbiological Determinations

Each risotto sample (10 g) was aseptically transferred into a sterile filter stomacher bag (BagPage, Brussels, Belgium) containing 90 mL of sterile Ringer’s solution (Oxoid, Barcelona, Spain). After homogenization for 2 min using a Stomacher 400 (Seward, London, UK), serial decimal dilutions were prepared for inoculation onto the culture media described below. The growth of the natural microbiota and C. perfringens was monitored using two selective culture media. Plate Count Agar (PCA) (Conda, Madrid, Spain) was used to enumerate aerobic mesophilic bacteria (AMB) after incubation at 30 °C for 24 h, while additional PCA plates were incubated at 5 °C for 10 days to quantify psychrophilic bacteria. *C. perfringens* was enumerated on Tryptone Sulfite Neomycin (TSN) agar (Conda, Madrid, Spain), which was incubated at 37 °C for 24 h under anaerobic conditions. In both cases, microorganisms were quantified using the pour plate technique, and the results were expressed as log CFU/g.

#### 2.4.4. pH Measurement

The pH of each sample was determined with a calibrated pH meter (micropH 2001, CRISON, Barcelona, Spain). The instrument was calibrated with standard phosphate buffer solutions at pH 4.0 and 7.0 before measurements were taken. Three readings were obtained at different locations within each sample, and the mean value was used for statistical analysis.

#### 2.4.5. Determination of Water Activity (Aw)

The water activity (Aw) of each sample was measured in triplicate with an AQUALAB 4TE water activity meter (Meter Group, Pullman, WA, USA).

#### 2.4.6. Lipid Oxidation Assessment

Lipid oxidation was assessed by determining thiobarbituric acid reactive substances (TBARS). A 10 g portion of the homogenized sample was mixed with 50 mL of distilled water using an Ultra-Turrax homogenizer (IKA T25 Basic, Staufen, Germany). Subsequently, 47.5 mL of distilled water and 2.5 mL of 4 N HCl (adjusted to pH 1.5) were added. After the addition of a few drops of antifoaming agent (Scharlau, Barcelona, Spain), the mixture was distilled (Foss Tecator, Höganäs, Sweden) until 50 mL of distillate had been collected. When necessary, the distillation was repeated to ensure complete recovery of the reaction products. An aliquot (5 mL) of the distillate was then mixed with an equal volume of 0.02 M thiobarbituric acid (TBA) solution in glass tubes. After thorough mixing, the tubes were incubated in a water bath at 96 °C for 40 min and then cooled to room temperature. Absorbance was measured at 538 nm using a spectrophotometer (Hitachi, Tokyo, Japan). TBARS concentrations were calculated from a calibration curve prepared with 1,1,3,3-tetraethoxypropane (TEP) and expressed as mg malondialdehyde (MDA)/kg of sample. It should be noted that the extraction solvents were completely removed by rotary evaporation and freeze-drying before the extracts were incorporated into the risotto (Section 2.1.2), so that the extracts were added as solvent-free powders. No residual solvent was therefore introduced into the food model, and the control treatment (C), prepared without extract, acted simultaneously as the vehicle control for the TBARS and for the remaining physicochemical determinations.

### 2.5. Statistical Analysis

The results are expressed as mean ± standard deviation (SD). Statistical analyses were performed with Statgraphics XVII-X64 (Statgraphics Technologies, Inc., The Plains, VA, USA). Treatment means were compared by one-way ANOVA, and significant differences were identified using Tukey’s multiple comparison test at *p* < 0.05.

## 3. Results and Discussion

### 3.1. Antimicrobial Activity

The antimicrobial activity of the three *P. carpunya* was assessed against a panel of 20 bacterial strains, and the findings are summarized in Table 2.

Eight bacterial species were found susceptible, with inhibition zones ranging from 7 to 16 mm, and minimum inhibitory concentrations (MIC) ranging from 10 to 80 mg/mL (Table 3).

The ethanolic extract (ETOH) of *P. carpunya* exhibited the greatest antimicrobial activity, as shown by its ability to inhibit the growth of several bacterial strains, including *Bacillus cereus*, *Campylobacter jejuni*, *Clostridium perfringens*, *Listeria innocua*, *Shewanella putrefaciens*, *Shewanella* sp. and *Yersinia enterocolitica* (Table 2 for a comprehensive list of tested strains). This biological activity is most likely related to the high levels of volatile compounds reported for *P. carpunya*, for the extraction of which ethanolic solvents have proven highly efficient [48,49]. Compounds such as 1,8-cineole [50] and safrole [51,52,53], both predominant within the genus *Piper*, and the antimicrobial activity linked to piperitone [26] and to other monoterpenes such as α-pinene and β-pinene [26] in other *Piper* species [54,55], provide a plausible explanation for the differences observed between solvents. Because the chemical composition of the extracts was not determined in the present work, these attributions should be regarded as hypotheses derived from the literature rather than as mechanisms demonstrated here.

The ethanolic extracts (ETOH) showed MIC values of 80 mg/mL against *B. cereus* and *L. innocua*, 40 mg/mL against *Y. enterocolitica*, 20 mg/mL against *C. jejuni*, *S. putrefaciens* and *Shewanella* sp., and 10 mg/mL against *C. perfringens* (see Table 3). The freeze-dried extract showed MIC values of 80 mg/mL against *B. thermosphacta* and *C. perfringens*, and 40 mg/mL against *B. cereus*. In contrast, ETOH–H_2_O extracts exhibited MIC values of 80 mg/mL against *B. thermosphacta* and *C. jejuni*, 40 mg/mL against *Y. enterocolitica*, and 20 mg/mL against *C. perfringens*. These MIC values are relatively high compared with those reported for the essential oil of *P. carpunya* by Valarezo et al. [26], who reported MIC values of 500 and 1000 µg/mL for *K. pneumoniae* and *E. faecalis,* respectively; this difference is expected, since essential oils concentrate the volatile fraction, whereas the solvent extracts used here also contain non-volatile matrix components. Additionally, these values are similar to those reported by Rondón et al. [25], namely 100 µg/mL for *S. aureus*, 200 µg/mL for *E. coli*, 300 µg/mL for *K. pneumoniae*, 800 µg/mL for *P. aeruginosa*, and 900 µg/mL for *E. faecalis*. Furthermore, ethanolic extracts of *P. carpunya* at a concentration of 25 mg/mL were able to inhibit the growth of *S. aureus*, *Staphylococcus epidermidis*, *Bacillus subtilis*, *B. cereus*, and *Candida albicans* [56]. In contrast, methanolic extracts exhibited substantial antibacterial activity against *E. coli* and mild activity against *Pseudomonas aeruginosa* [28].

Quilez et al. [21] reported that *P. carpunya* exhibits antimicrobial activity attributed to its secondary metabolites mainly against *H. pylori*. Bioactive compounds, including flavonoids, terpenoids, neolignans, and dihydrochalcones, have been isolated from *P. carpunya.* Among the flavonoids isolated from *P. carpunya* are vitexin, isovitexin, and 4′-O-methyl-2″-O-α-L-rhamnopyranosylvitexin have been isolated from *P. carpunya* [21]. These compounds are recognized for their potent antioxidant and free radical scavenging activities, which may contribute to their antibacterial effects. Vitexin has been shown to interfere with *S. aureus* biofilm development by altering the surface hydrophobicity of the bacterium [57]. Furthermore, neolignan (6S, 9S)-roseoside, which has also been identified in *P. carpunya* [21], was isolated by Wu et al. [58] from *Senecio cannabifolius* Less. This compound showed notable antibacterial activity against *S. aureus* and *B. subtilis*, with MIC values ranging from 30 to 60 μg/mL.

Furthermore, terpenoids, including *β*-sitosterol and stigmasterol, which are present in *P. carpunya* [21], have been isolated from the stem extract of *Cissus populnea* [59], and β-sitosterol from *Odontonema strictum* showed significant antimicrobial activity. β-sitosterol and stigmasterol showed antimicrobial activity against methicillin-resistant *S. aureus* (MRSA), *S. aureus*, *E. coli*, *C. albicans*, *Trichophyton rubrum*, and *Trichophyton mentagrophyte*. Finally, the dihydrochalcone asebogenin isolated from *P. carpunya* [21] was also identified by Orjala et al. [60] in *Piper aduncum* and is known for its antibacterial activity against *B. subtilis*, *Micrococcus luteus*, and *E. coli*. Considered together, these reports suggest that *P. carpunya* contains constituents with broad-spectrum antimicrobial activity and support its potential development as a source of antimicrobial agents; it should nevertheless be stressed that these compounds were not quantified in the extracts assayed here, so their individual contribution to the activity reported below cannot be established.

To date, no research has evaluated the application of *P. carpunya* extract as a food additive. Although its antioxidant and antimicrobial properties are recognized in ethnomedicinal contexts [32], scientific attention has not yet turned to its functional integration into food systems. Notably, although the plant is traditionally utilized in the preparation of *Guaviduca*—a beverage from the Ecuadorian Amazon produced by the Shuar community [26]—the absence of studies regarding its direct application to food matrices underscores the significance of this research. To address this gap in the literature, the second phase of this study aimed to assess the inhibitory effect of *P. carpunya* extracts on the growth of *C. perfringens* in a risotto-based food model.

### 3.2. Effect of P. carpunya Extracts on Microbial Growth of Clostridium Perfringens in Risotto During Storage

*Piper carpunya* extracts—particularly the hydroethanolic fraction—were previously reported by Reyes et al. [61] at the XII National CyTA-CESIA Congress to exhibit a high total phenolic content and exceptional in vitro antioxidant capacity, as determined by DPPH, FRAP, and ABTS assays; a comparative characterization of *P. carpunya* extracts obtained by four extraction methods has also been reported by our group [37]. These preliminary findings provided the rationale for evaluating the effect of these extracts on the microbial growth of *C. perfringens* in a risotto model during storage.

*C. perfringens* was selected for this study because of its sensitivity to the three extracts evaluated (ETOH, ETOH-H_2_O, and L) and because it is a major enterotoxin-producing pathogen responsible for foodborne intoxications [62]. This Gram-positive, anaerobic, spore-forming microorganism has a broad growth range (12 °C to 50 °C), with an optimal temperature between 43 °C and 47 °C [63]. Notably, its growth is exceptionally rapid, with a generation time of only 7.1 min at 41 °C [63]. As a causative agent of various histotoxic and gastrointestinal diseases in both humans and livestock, *C. perfringens* strains can produce at least 20 different toxins [64]. The most virulent include α (CPA), β2 (CPB2), ε (ETX), and ι (ITX) [65], together with the enterotoxin CPE. The latter is a primary cause of food poisoning and ranks as the third most common foodborne illness in the United States, with an estimated one million cases annually [62,66]. Although typically non-lethal, the intoxication manifests 8–10 h after ingestion, causing symptoms such as acute diarrhea and abdominal pain [67].

This stage of the investigation sought to determine whether the effects of *P. carpunya* extracts observed in vitro could be reproduced in a precooked risotto food model. The antimicrobial potential of the extracts was evaluated by assessing their inhibitory effects on the growth of *C. perfringens* and aerobic mesophilic bacteria (AMB) within the risotto matrix. As shown by the data presented in Table 4, the non-inoculated samples (control [C], lyophilized [L], and hydroethanolic [E]) exhibited no growth of *C. perfringens*.

Conversely, inoculated treatments demonstrated initial counts of approximately 5 log CFU/g on day 0, attributable to the inoculum. The extracts exhibited antimicrobial properties, evidenced by a substantial decrease in bacterial counts in the EP and LP treatments relative to the P control from day 0. This reduction persisted until day 21 for EP, whereas LP no longer differed significantly from P by day 1, indicating a lower antimicrobial effect of the lyophilized extract (L). Although the differences in LP were not significant until day 7, lower values were nonetheless observed relative to the control. The EP treatment showed a significant reduction in *C. perfringens* counts—approximately 2 log units—by day 7 compared with control P, and counts fell below the detection limit from day 15 onwards. This inhibitory effect was consistent with the in vitro results and may be related to the greater efficacy of hydroethanolic mixtures (E) in extracting phenolic compounds [68], whereas aqueous extracts have been shown to yield higher amounts of polysaccharides, proteins, and active peptides [49]. Constituents reported in the essential oils of *P. carpunya*, such as 1,8-cineole, safrole, and piperitone, α-pinene, and β-pinene [26], which are characteristic of the genus *Piper* [35], are plausible contributors to the bioactivity observed.

Furthermore, a substantial decrease in *C. perfringens* counts was observed in the inoculated treatments (P, EP, and LP) during storage, most likely attributable to the refrigeration conditions, which suppressed bacterial growth. Research has shown that *C. perfringens* is unable to survive at temperatures below 10 °C [69,70], a phenomenon that may be attributed to the absence of unsaturated fatty acid synthesis as a mechanism of adaptation [69].

### 3.3. Effect of P. carpunya Extracts on Microbial Growth of Aerobic Mesophilic Bacteria (AMB) in Risotto During Storage

Table 5 presents the AMB counts, which serve as an indicator of bacterial spoilage and product shelf life [71,72]. A significant increase in colony counts was observed during the storage period, reaching 3.0–6.0 log units by the final day of analysis. The hydroethanolic (ETOH–H_2_O) extract (E) exhibited antibacterial activity, with significantly lower AMB counts than the control (C) from day 15 onwards, maintaining a reduction of 2 to 4 log CFU/g until the end of the study. In the EP treatment, counts remained significantly lower than in the P treatment from day 1 to day 4; thereafter, however, the efficacy of the extract was attenuated, potentially owing to interactions with food components [45,46] such as lipids, proteins, and carbohydrates [47]. Despite this, slight differences persisted, particularly on day 21. Notably, the L and LP treatments began the study (day 0) with high AMB counts (4.57 and 5.31 log CFU/g), and on the remaining days they showed values similar to those of the P treatment, most likely as a result of cross-contamination during the extraction process. This observation indicates that the freeze-dried extract itself contributed a microbial load to the product and that a decontamination step would be required before any practical use.

The analysis of psychrophilic bacterial growth, as presented in Table 6, revealed patterns comparable to those observed for AMB. All treatments resulted in a significant increase in colony counts over the storage period. Notably, treatments C and P reached counts between 6 and 7 log CFU/g by day 30, exceeding the values for the other treatments and those recorded for AMB. This increase is likely due to refrigeration conditions that favored psychrophilic proliferation; these microorganisms can thrive at low temperatures through molecular and morphological adaptations [73,74]. Treatments L and LP initially exceeded 3 log CFU/g, again consistent with contamination of the freeze-dried extract. Final counts for treatments E, L, EP, and LP ranged between 4 and 5 log CFU/g and were therefore lower than those for C and P, indicating antimicrobial effects of the extracts. The hydroethanolic extract (E) treatments showed significantly lower counts than L, P, and LP up to day 21, with a reduction of 2 to 3 log units. In addition, the EP treatment showed a significant decrease of approximately 2 log CFU/g relative to P on day 7 and 21, which is consistent with the antimicrobial activity demonstrated in vitro, although the difference was not statistically significant at day 15 because of the variability of the counts.

### 3.4. Effect of Freeze-Dried and Ethanol-Water Extracts of P. carpunya on the pH of Risotto During Storage

The pH of food products is a critical factor in determining safety, since changes in pH can serve as indicators of spoilage driven by chemical and microbial reactions. Furthermore, pH exerts a significant influence on the sensory and textural characteristics of the food matrix [75]. As shown in Table 7, the extract-containing treatments remained stable over the storage period, with no significant changes over time, whereas the control showed a slight but significant decrease between day 4 and day 30. Treatments containing extracts (L and E) had significantly lower pH values than the control (C) on day 0, and the hydroethanolic extract (E) maintained this difference up to day 30. This may be attributed to the presence of organic acids, such as phenolic acids [37], in the extracts of *P. carpunya*.

The addition of *P. carpunya* extract to the food matrix generally caused a statistically significant decrease in pH compared with the control (Table 7). This reduction was less pronounced in the lyophilized extract (L) treatment, whereas the hydroethanolic extract (E) caused the greatest pH decrease. Statistically significant differences were therefore observed according to the type of extract rather than according to storage period. These differences and trends remained consistent across all treatments during the storage period. The decrease in pH may be ascribed to the presence of phenolic compounds [35]; moreover hydroalcoholic solutions are known to enhance the extraction of such compounds [76,77], which would explain the lower pH values observed in the samples containing the hydroethanolic extract. The lyophilized extract also caused a slight decrease in pH, likely due to the extraction of certain phenolic compounds, albeit to a lesser extent.

### 3.5. Effect of Freeze-Dried and Ethanol–Water Extracts of P. carpunya on the Water Activity (Aw) of Risotto During Storage

Water activity (*Aw*) is an intrinsic property of foods that affects texture, flavor, color, taste, nutritional value, and shelf life, since it either facilitates or inhibits microbial growth [78,79]. During the 30-day storage period, significant differences in the *Aw* of risotto (Table 8) were observed between the treatments containing *P. carpunya* extracts (hydroethanolic [E] and lyophilized [L]) and the control (C).

In general, from the initial day of the experiment, treatments with E and L exhibited significantly lower *Aw* values than the control group (*p* < 0.05), suggesting an immediate effect of the extracts on reducing water availability in the product. This trend remained constant until day 7 and was again significant at day 30, highlighting the efficacy of the extracts in sustaining lower Aw levels. It is noteworthy that no significant variation in Aw was detected within each treatment throughout the observation period.

### 3.6. Effect of Freeze-Dried and Ethanol–Water Extracts of P. carpunya on the Oxidation of Risotto During Storage

Lipid oxidation induces modifications in lipids through the formation of free radicals in a series of chain reactions, adversely affecting the organoleptic and nutritional properties of foods. Oxidative stability refers to the resistance to the formation of these radicals [80]. Malondialdehyde (MDA), a secondary product of lipid peroxidation [81], is undesirable in food products as it forms colored compounds when reacting with thiobarbituric acid (TBA). This method relies on the reaction of MDA with TBA to measure secondary oxidation products, expressed as mg of MDA/kg, with higher values indicating greater oxidation. According to some authors, malondialdehyde levels above 2 mg/kg in meat systems are within the range at which rancidity is detectable by consumers [82]. Furthermore, the flavonoids present in the extracts might shift from antioxidant to pro-oxidant behavior depending on their concentration and on the composition of the matrix, which could explain varying effects observed in different treatments [83,84].

The malondialdehyde (MDA) content in risotto, evaluated as a marker of secondary lipid oxidation, showed distinct patterns among treatments during the 30-day storage period (Table 9). Throughout the study, both the hydroethanolic (E) and lyophilized (L) extract treatments consistently maintained higher MDA concentrations than the control group (C). This behavior indicates a prooxidant rather than an antioxidant effect of the extracts within the food matrix, in contrast to the in vitro antioxidant capacities previously reported by Guayllas et al. [76], and to the strong positive correlation between total phenolic content, antimicrobial activity, and antioxidant capacity described in the literature [36,68,85]. Such a reversal is well documented for polyphenols, which can act as prooxidants when they are present at high concentrations or in the presence of transition metals [80,83]; the dose applied here (8%, w/w) is high, and the risotto contains dairy ingredients, so both conditions may have been met. This interpretation remains tentative, however, because neither the phenolic content of the extracts nor the metal content of the matrix was measured in this study.

However, from a food quality and stability perspective, it is noteworthy that MDA levels in all samples remained exceptionally low over time. The maximum concentration recorded was 0.32 ± 0.10 mg MDA/kg (hydroethanolic treatment E, Days 7 and 15), which is safely below the widely accepted threshold of 2.0 mg MDA/kg typically associated with rancidity and quality rejection in fat-containing food systems [86]. Therefore, despite the statistical differences observed among treatments as a result of the extract additions, the chemical quality was preserved throughout the 30 days of storage.

On the fourth day of the experiment, MDA levels increased marginally in all treatment groups; however, levels in the control (C) remained significantly lower than those in E and L. By the seventh day, while lipid oxidation had progressed in all samples, differences between treatments were no longer statistically significant, suggesting a possible convergence of oxidative processes. This pattern persisted on day 15, with no significant differences observed among treatments despite the continued presence of elevated MDA levels in hydroethanolic (E) and lyophilized (L) samples. Finally, on day 30, the control group exhibited a marked decrease in MDA (0.03 ± 0.03 mg MDA/kg), which was significantly lower than the levels observed in E and L (0.29 ± 0.02 and 0.23 ± 0.01 mg MDA/kg, respectively). These results suggest that, rather than exerting an antioxidant effect, *P. carpunya* extracts contributed to an increase in lipid oxidation throughout most of the storage period, although the values reached remained well below the rancidity thresholds reported in the literature [87,88]. Furthermore, the control group presented low MDA values (<1 mg/kg) throughout the storage period, reflecting the remarkable oxidative stability of the risotto matrix.

### 3.7. Sensory Implications, Limitations of the Study, and Future Research

An aspect that must be considered before any practical application is the sensory impact of the extract. The dose used here (8%, *w*/*w*) was selected to guarantee an antimicrobial effect inside the food matrix and is high relative to the levels normally applied for plant-derived preservatives. *P. carpunya* is an aromatic species whose leaves and essential oil are dominated by volatile constituents such as 1,8-cineole, safrole, piperitone, α-pinene, and β-pinene [26], and it is traditionally consumed as the infusion known as Guaviduca precisely because of its aroma [19,26]. It is therefore reasonable to expect that its incorporation at 8% (*w*/*w*) modifies the aroma and flavor of a mild matrix such as risotto, as has repeatedly been described for essential oils and phenolic-rich extracts, whose intense odor is one of the main obstacles to their use as food preservatives [89]. In a comparative study of essential oils and conventional preservatives in vegetable sauces, the antimicrobial benefit obtained was likewise conditioned by consumer acceptance [90]. No sensory evaluation was carried out in the present work, so no claim can be made regarding the acceptability of the treated risotto. The physicochemical data do show, however, that the extracts did not generate off-flavors of oxidative origin, since malondialdehyde remained below 0.35 mg/kg throughout storage, far below the values associated with perceptible rancidity [82,86]. Strategies such as microencapsulation, nanoemulsification, or the combination of lower extract doses with additional hurdles (reduced water activity, modified atmosphere, or mild thermal treatment) would make it possible to lower the antimicrobial dose and limit the sensory impact [89], and they constitute the natural continuation of this work.

## 4. Conclusions

This study establishes *P. carpunya* as a promising source of natural antimicrobial agents for the food industry and translates its traditional use in the Ecuadorian Amazon into a food-technology context. Because the scientific information available is limited, this work represents one of the first attempts to evaluate *P. carpunya* extracts inside a real food matrix rather than only in vitro. Our data show that *P. carpunya* extracts exhibit antibacterial activity against *B. cereus*, *L. innocua*, *B. thermosphacta*, *Y. enterocolitica*, *Shewanella* sp., *S. putrefaciens*, *C. perfringens*, and *C. jejuni*. Among the extracts studied, the ethanol extract was the most effective in vitro but had a very low yield (1.71%), followed by the hydroethanolic and the lyophilized extracts. In the risotto model, the hydroethanolic extract reduced *C. perfringens* counts from day 1 to day 21 and aerobic mesophilic counts up to day 4, and it also lowered psychrophilic counts. The lyophilized extract was less effective, showing lower bacterial counts only up to day 4 of storage and no significant difference in aerobic mesophilic or psychrophilic counts compared with the inoculated control. These benefits must, however, be weighed against two findings that temper the practical use of the extracts: both extracts lowered the pH and the water activity of the product relative to the control, and, instead of protecting the lipid fraction, they exerted a prooxidant effect throughout most of the storage period, although malondialdehyde values remained below 0.35 mg/kg and therefore well below the thresholds associated with perceptible rancidity. In addition, the compounds responsible for these effects could not be identified, since the extracts were not chemically characterized in this study, and the sensory impact of the dose applied (8%, *w*/*w*) was not evaluated. *P. carpunya* extracts can therefore be regarded as promising natural ingredients for improving the microbiological safety of ready-to-eat foods, provided that future work characterizes the extracts by LC-MS/MS or GC-MS, correlates their constituents with the biological activities observed, optimizes the dose through encapsulation or hurdle strategies, and confirms their sensory acceptability in the target matrix.

## Figures and Tables

**Table 1:** Bacterial strains used in the antimicrobial assays and their culture conditions.

**Microorganism**	**Reference Strain**	**Culture Conditions**	**Assay Medium**
*Aeromonas caviae*	CECT 838	TSB, 30 °C	Muller Hinton
*Bacillus cereus*	CECT 148	BHI, 30 °C	Muller Hinton
*Brochothrix thermosphacta*	CECT 847	TSB + Yeast 0.6%, 26 °C	Muller Hinton
*Campylobacter jejuni*	CECT 7572	BHI, 42 °C	Nutrient + 5% blood
*Clostridium perfringens*	CECT 376	BHI, 42 °C	Nutrient + 5% blood
*Enterococcus faecalis*	CECT 481	BHI, 37 °C	Nutrient + 5% blood
*Escherichia coli*	CECT 501	BHI, 37 °C	Muller Hinton
*Leuconostoc mesenteroides*	CECT 394	MRS, 30 °C	MRS
*Listeria innocua*	CECT 910	BHI, 37 °C	Muller Hinton
*Listeria monocytogenes*	ILSI-4	BHI, 37 °C	Muller Hinton
*Pseudomonas fluorescens*	CECT 378	TSB, 30 °C	Muller Hinton
*Pseudomonas putida*	CECT 3241	TSB, 30 °C	Muller Hinton
*Salmonella enterica* subsp. *enterica*	HUBU-UBU 72732	TSB, 37 °C	Muller Hinton
*Shewanella putrefaciens*	CECT 5346	TSB, 30 °C	Muller Hinton
*Shewanella* sp.	CECT 4640	TSB, 30 °C	Muller Hinton
*Shigella sonnei*	CECT 457	TSB, 37 °C	Muller Hinton
*Staphylococcus aureus*	CECT 30	BHI, 37 °C	Muller Hinton
*Vibrio alginolyticus*	CECT 521	Nutrient broth + 2% NaCl, 30 °C	Nutrient agar + 2% NaCl
*Weissella viridescens*	CECT 283	MRS, 30 °C	MRS
*Yersinia enterocolitica*	CECT 559	TSB, 37 °C	Muller Hinton

CECT, Spanish Type Culture Collection; ILSI: International Life Science Institute; HUBU-UBU: Hospital of Burgos-University of Burgos.

**Table 2:** Inhibition zone diameters (mm) produced by *P. carpunya* extracts against the tested microorganisms.

Microorganism	Extracts			
	ETOH	L	ETOH–H_2_O	Positive Control
*Aeromonas caviae*	ND	ND	ND	20.0
*Bacillus cereus*	8.0	9	ND	17.0
*Brochothrix thermosphacta*	ND	7.0	8.0	24.0
*Campylobacter jejuni*	15.0	7.0	8.0	15.0
*Clostridium perfringens*	13.0	10.0	16.0	11.0
*Enterococcus faecalis*	ND	ND	ND	11.0
*Escherichia coli*	ND	ND	ND	17.0
*Leuconostoc mesenteroides*	ND	ND	ND	19.0
*Listeria innocua*	9.0	ND	ND	13.0
*Listeria monocytogenes*	ND	ND	ND	16.0
*Pseudomonas fluorescens*	ND	ND	ND	19.0
*Pseudomonas putida*	ND	ND	ND	16.0
*Salmonella enterica* subsp. *enterica*	ND	ND	ND	15.0
*Shewanella putrefaciens*	8.0	ND	ND	27.0
*Shewanella* sp.	9.0	ND	ND	29.0
*Shigella sonnei*	ND	ND	ND	16.0
*Staphylococcus aureus*	ND	ND	ND	13.0
*Vibrio alginolyticus*	ND	ND	ND	16.0
*Weissella viridescens*	ND	ND	ND	21.0
*Yersinia enterocolitica*	9.0	ND	ND	19.0

Values are expressed as inhibition zone diameter (mm). ND: no inhibition detected. Positive control: sodium hypochlorite (0.94%). ETOH: ethanolic extract; ETOH–H_2_O: hydroethanolic extract (50:50, *v*/*v*); L: freeze-dried aqueous extract.

**Table 3:** Minimum inhibitory concentrations (MICs, mg/mL) of *P. carpunya* extracts against susceptible bacteria.

Microorganism	Extracts		
	ETOH	L	ETOH–H_2_O
*Bacillus cereus*	80	40	ND
*Brochothrix thermosphacta*	ND	80	80
*Campylobacter jejuni*	20	ND	80
*Clostridium perfringens*	10	80	20
*Listeria innocua*	80	ND	ND
*Shewanella putrefaciens*	20	ND	ND
*Shewanella* sp.	20	ND	ND
*Yersinia enterocolitica*	40	ND	40

The concentration range evaluated was 5–80 mg/mL. ND: no inhibition detected within the concentration range tested. ETOH: ethanolic extract; ETOH–H_2_O: hydroethanolic extract (50:50, *v*/*v*); L: freeze-dried aqueous extract. Values are representative of three independent determinations (*n* = 3).

**Table 4:** Changes in *C. perfringens* counts (log CFU/g) in the risotto model during refrigerated storage.

	Treatments					
Days	C	L	E	P	LP	EP
**0**	ND	ND	ND	5.28 ± 0.11 ^a,A^	4.69 ± 0.17 ^c,A^	4.99 ± 0.14 ^b,A^
**1**	ND	ND	ND	4.74 ± 0.23 ^a,AB^	4.15 ± 0.11 ^a,A^	2.99 ± 0.40 ^b,B^
**4**	ND	ND	ND	4.31 ± 0.32 ^a,BC^	3.43 ± 0.19 ^b,AB^	2.25 ± 0.37 ^c,BC^
**7**	ND	ND	ND	3.67 ± 0.17 ^a,C^	2.36 ± 0.25 ^ab,BC^	1.72 ± 1.50 ^b,BCD^
**15**	ND	ND	ND	1.55 ± 0.28 ^a,D^	1.62 ± 0.27 ^a,CD^	ND
**21**	ND	ND	ND	ND	1.19 ± 1.04 ^CD^	ND

Values are expressed as mean ± SD (*n* = 3). a–c Different letters within the same row indicate significant differences among treatments. A–D Different letters within the same column indicate significant differences over storage time (*p* < 0.05). ND: not detected (<1 log CFU/g). C: without extract; L: containing freeze-dried extract; E: containing the hydroethanolic extract (ETOH–H_2_O); P: inoculated control; LP: inoculated risotto containing the freeze-dried extract; EP: inoculated risotto containing the hydroethanolic extract (ETOH–H_2_O).

**Table 5:** Changes in aerobic mesophilic bacterial (AMB) counts (log CFU/g) in the risotto model during refrigerated storage.

	Treatments					
Days	C	L	E	P	LP	EP
0	ND	4.57 ± 0.15 ^b,BC^	ND	ND	5.31 ± 0.03 ^a,ABC^	1.30 ± 0.00 ^c,CD^
1	ND	4.44 ± 0.17 ^a,BC^	ND	3.55 ± 0.21 ^a,C^	4.17 ± 1.10 ^a,C^	1.36 ± 0.32 ^b,CD^
4	ND	4.20 ± 0.28 ^a,C^	1.73 ± 0.26 ^b,ABC^	4.14 ± 0.11 ^a,BC^	4.08 ± 0.14 ^a,C^	1.00 ± 0.00 ^c,D^
7	1.26 ± 1.10 ^b,B^	4.87 ± 0.14 ^a,AB^	3.03 ± 0.63 ^ab,A^	4.72 ± 0.00 ^ab,ABC^	4.58 ± 0.10 ^a,BC^	2.80 ± 0.70 ^ab,BC^
15	6.60 ± 0.17 ^a,A^	4.81 ± 0.30 ^abc,AB^	2.18 ± 1.38 ^c,AB^	5.14 ± 1.55 ^ab,ABC^	5.58 ± 0.20 ^ab,AB^	3.45 ± 0.87 ^bc,B^
21	5.77 ± 0.32 ^a,A^	4.86 ± 0.11 ^ab,AB^	3.57 ± 0.79 ^c,A^	5.95 ± 0.40 ^a,A^	5.84 ± 0.40 ^a,AB^	4.15 ± 0.13 ^bc,B^
30	5.34 ± 0.26 ^ab,A^	5.19 ± 0.08 ^ab,A^	3.18 ± 0.46 ^c,A^	5.38 ± 0.28 ^ab,AB^	5.85 ± 0.06 ^a,A^	4.31 ± 1.09 ^bc,B^

Values are expressed as mean ± SD (*n* = 3). a–c Different letters within the same row indicate significant differences among treatments. A–D Different letters within the same column indicate significant differences over storage time (*p* < 0.05). ND: not detected (<1 log CFU/g). C: without extract; L: containing freeze-dried extract; E: containing the hydroethanolic extract (ETOH–H_2_O); P: inoculated control; LP: inoculated risotto containing the freeze-dried extract; EP: inoculated risotto containing the hydroethanolic extract (ETOH–H_2_O).

**Table 6:** Changes in psychrophilic bacterial counts (log CFU/g) in the risotto model during refrigerated storage.

	Treatments					
Days	C	L	E	P	LP	EP
0	ND	3.21 ± 0.21 ^a,B^	ND	ND	3.64 ± 0.10 ^a,BC^	ND
1	1.05 ± 0.91 ^b,C^	3.48 ± 0.18 ^a,B^	ND	ND	2.14 ± 1.61 ^ab,C^	1.00 ± 0.00 ^bc,C^
4	2.78 ± 1.41 ^ab,BC^	3.53 ± 0.54 ^a,B^	ND	1.90 ± 0.17 ^ab,C^	3.41 ± 0.52 ^a,BC^	1.40 ± 0.37 ^bc,BC^
7	3.45 ± 1.98 ^ab,ABC^	3.42 ± 0.10 ^ab,B^	ND	3.98 ± 0.00 ^a,B^	2.86 ± 0.14 ^ab,BC^	1.26 ± 0.24 ^b,BC^
15	4.12 ± 2.54 ^ab,ABC^	4.05 ± 0.39 ^ab,B^	2.21 ± 0.96 ^b,B^	6.06 ± 0.00 ^a,A^	3.75 ± 0.65 ^ab,ABC^	4.18 ± 0.00 ^ab,BC^
21	5.45 ± 0.46 ^a,AB^	5.40 ± 0.70 ^a,A^	2.99 ± 0.41 ^b,AB^	5.45 ± 1.13 ^a,A^	5.60 ± 0.22 ^a,A^	2.59 ± 0.78 ^b,B^
30	7.23 ± 0.32 ^a,A^	4.34 ± 0.78 ^a,AB^	4.35 ± 1.16 ^a,A^	6.15 ± 0.00 ^a,A^	4.85 ± 1.60 ^a,AB^	4.35 ± 0.50 ^a,A^

Values are expressed as mean ± SD (*n* = 3). a–c Different letters within the same row indicate significant differences among treatments. A–C Different letters within the same column indicate significant differences over storage time (*p* < 0.05). ND: not detected (<1 log CFU/g). C: without extract; L: containing freeze-dried extract; E: containing the hydroethanolic extract (ETOH–H_2_O); P: inoculated control; LP: inoculated risotto containing the freeze-dried extract; EP: inoculated risotto containing the hydroethanolic extract (ETOH–H_2_O).

**Table 7:** Changes in the pH of the precooked risotto model during refrigerated storage at 5 °C for 30 days.

	Treatments		
Days	C	E	L
0	7.00 ± 0.06 ^a,AB^	6.43 ± 0.04 ^c,A^	6.73 ± 0.04 ^b,A^
1	6.95 ± 0.03 ^a,AB^	6.48 ± 0.06 ^b,A^	6.80 ± 0.19 ^a,A^
4	7.07 ± 0.01 ^a,A^	6.42 ± 0.16 ^b,A^	6.77 ± 0.08 ^ab,A^
7	6.95 ± 0.06 ^a,AB^	6.32 ± 0.00 ^c,A^	6.70 ± 0.02 ^b,A^
15	6.97 ± 0.08 ^a,AB^	6.36 ± 0.04 ^c,A^	6.68 ± 0.05 ^b,A^
30	6.83 ± 0.00 ^a,B^	6.40 ± 0.13 ^b,A^	6.71 ± 0.04 ^a,A^

Values are expressed as mean ± SD (*n* = 3). a–c Different letters within the same row indicate significant differences among treatments. A–B Different letters within the same column indicate significant differences over storage time (*p* < 0.05). C: without extract; L: containing freeze-dried extract; E: containing the hydroethanolic extract (ETOH–H_2_O).

**Table 8:** Changes in water activity (Aw) of the precooked risotto model during refrigerated storage at 5 °C for 30 days.

	Treatments		
Days	C	E	L
0	0.995 ± 0.005 ^a,A^	0.977 ± 0.000 ^b,A^	0.976 ± 0.002 ^b,A^
1	0.997 ± 0.000 ^a,A^	0.975 ± 0.001 ^b,A^	0.978 ± 0.001 ^b,A^
4	0.997 ± 0.000 ^a,A^	0.978 ± 0.001 ^b,A^	0.977 ± 0.005 ^b,A^
7	0.996 ± 0.001 ^a,A^	0.974 ± 0.004 ^b,A^	0.973 ± 0.003 ^b,A^
15	0.994 ± 0.001 ^a,A^	0.976 ± 0.011 ^a,A^	0.976 ± 0.006 ^a,A^
30	0.996 ± 0.002 ^a,A^	0.980 ± 0.004 ^b,A^	0.982 ± 0.002 ^b,A^

Values are expressed as mean ± SD (*n* = 3). a–b Different letters within the same row indicate significant differences among treatments; identical capital letters in the same column indicate no significant differences over the storage period (*p* < 0.05). C: without extract; L: containing freeze-dried extract; E: containing the hydroethanolic extract (ETOH–H_2_O).

**Table 9:** Changes in malondialdehyde (MDA) content (mg/kg) in the risotto model during refrigerated storage at 5 °C for 30 days.

Samples		C	E	L
Days	0	0.02 ± 0.02 ^b,A^	0.20 ± 0.05 ^a,A^	0.13 ± 0.01 ^ab,A^
	1	0.01 ± 0.00 ^b,A^	0.23 ± 0.00 ^a,A^	0.19 ± 0.02 ^a,A^
	4	0.03 ± 0.02 ^b,A^	0.27 ± 0.02 ^a,A^	0.18 ± 0.02 ^a,A^
	7	0.08 ± 0.10 ^a,A^	0.32 ± 0.10 ^a,A^	0.22 ± 0.14 ^a,A^
	15	0.07 ± 0.09 ^a,A^	0.32 ± 0.13 ^a,A^	0.26 ± 0.12 ^a,A^
	30	0.03 ± 0.03 ^b,A^	0.29 ± 0.02 ^a,A^	0.23 ± 0.01 ^a,A^

Values are expressed as mean ± SD (*n* = 3). a–b Different letters within the same row indicate significant differences among treatments. A Different letters within the same column indicate significant differences over storage time, (*p* < 0.05). C: without extract; L: containing freeze-dried extract; E: containing the hydroethanolic extract (ETOH–H_2_O).

## Data Availability

The original contributions presented in this study are included in the article. Further inquiries can be directed to the corresponding author.
